# Editorial: Obsessive-compulsive disorder (OCD) across the lifespan: Current diagnostic challenges and the search for personalized treatment

**DOI:** 10.3389/fpsyt.2022.927184

**Published:** 2022-08-22

**Authors:** Roseli G. Shavitt, Odile A. van den Heuvel, Christine Lochner, Y. C. Janardhan Reddy, Euripedes C. Miguel, Helen Blair Simpson

**Affiliations:** ^1^Department of Psychiatry, Faculdade de Medicina FMUSP, Universidade de São Paulo, São Paulo, Brazil; ^2^Department of Psychiatry, Amsterdam UMC, Vrije Universiteit Amsterdam, Amsterdam, Netherlands; ^3^Department of Anatomy and Neurosciences, Amsterdam UMC, Vrije Universiteit Amsterdam, Amsterdam, Netherlands; ^4^SA Medical Research Council Unit on Risk and Resilience in Mental Disorders, Department of Psychiatry, Stellenbosch University, Stellenbosch, South Africa; ^5^National Institute of Mental Health and Neurosciences (NIMHANS), Bangalore, India; ^6^New York State Psychiatric Institute, New York, NY, United States; ^7^Department of Psychiatry, Vagelos College of Physicians and Surgeons, Columbia University Irving Medical Center, New York, NY, United States

**Keywords:** obsessive-compulsive disorder, OCD phenotype, OCD neuropsychology, OCD neuroimaging, OCD genetics, OCD comorbidity

Obsessive-compulsive disorder (OCD) has an estimated worldwide lifetime prevalence ranging from 1 to 2.3%, which makes it twice as common as schizophrenia. It is a potentially incapacitating neuropsychiatric disorder, presenting with comorbid disorders in the vast majority of cases and an unsatisfactory response to conventional, first-choice treatments in approximately 30% of patients (1). The age of onset of OCD symptoms is commonly in childhood or adolescence, and currently a set of different symptom dimensions have been identified. The course of OCD is usually chronic, and, more rarely, symptoms follow an episodic pattern (2). The disease burden is further complicated by the worldwide well documented delay in establishing a correct diagnosis and initiating adequate treatment (3). In contrast, recent advances in the field revealed abnormalities in the neurocircuitry associated with OCD. Moreover, the resemblance of OCD symptoms across countries and cultures allows for the use of modern biotechnology and biomedicine to challenge the unmet needs of patients with OCD across the lifespan. In this context, in order to achieve more effective clinical care, further investigation of the factors associated with the diagnostic delay and the response to diverse treatment modalities across the lifespan is highly needed. Examples include the non-recognition of specific OCD symptoms, the non-recognition of environmental (e.g., trauma) and biological (e.g., genetics) risk factors for the development of the disorder, and the presence of comorbidities which have implications for the trajectory of OCD.

This collection presents 25 manuscripts, including original studies and reviews submitted by authors from all continents worldwide, conducted with samples of youth and adults with OCD. We present a brief comment on the articles, grouped by the main topics covered in this special issue: perinatal and life events, genetics, neuroimaging, neuropsychology, phenotype, comorbidity and treatment.

## Perinatal events, life events, developmental stage and OCD psychopathology

Ratzoni et al. investigated, in a non-clinical sample, the associations between the postnatal onset of parent-infant relationship OCD (PI-ROCD) symptoms and caregiver-infant interactions. First, the authors provided initial evidence for the postnatal onset of PI-ROCD symptoms and identify factors to be included in a future validation of a PI-ROCD screening measure. Then, they delineated a mediating mechanism for the longitudinal pathway of risk through which PI-ROCD symptoms might interfere with the ongoing reciprocal nature of interactions between mother and infant, emphasizing the need for early screening and planning of preventive interventions targeting maternal behaviors that may effectively moderate risk. The study addresses the cycle of preoccupation with the infant's future morality and competence reducing praising/increasing parental criticism, leading to the infant's avoidance of social engagement. This, in turn, may further increase parental fears and preoccupation with the child's morality and competence, reinforcing a vicious cycle. Therefore, targeting parental fears and preoccupations (beyond the anxiety symptoms typical in the postpartum period) with the future development of the child may promote a healthier parent-infant interaction.

The presence of maternal obsessive-compulsive symptoms (OCS) predicted maternal and offspring psychopathology in an original study by Blanco-Vieira et al. that included 2,511 mother-children dyads recruited from elementary schools. All obsessive-compulsive symptom dimensions detected in the mothers were associated with maternal psychopathology and, importantly, with higher rates of internalizing, externalizing and obsessive-compulsive symptoms in their offspring, with the different OCS dimensions having distinct associations with the Child Behavior Checklist domains (internalizing, externalizing and OCS domains). Such findings highlight the relevance of screening for maternal OCS as part of the implementation of preventive and early intervention strategies for adults with psychopathology and their children. A subsample from the same cohort comprising 378 children at high risk of developing a mental disorder was assessed by Salto et al. for clinical, genomic and structural neuroimaging data at two time points separated by 3 years. The authors aimed to explore the relationship between OCS and the rate of thalamic volume change over time. They found that a slower decrease in the right thalamic volume showed a positive relationship with the presence of OCS after 3 years.

The association of childhood maltreatment (CM) in its various forms (physical, sexual and emotional abuse and neglect) and the clinical symptomatology of OCD was addressed in a meta-analysis by Ou et al. Their results revealed that CM positively correlates with the severity of OCD, as well as depressive symptoms. In particular, emotional abuse correlated with the severity of obsessive-compulsive symptoms as a whole (total Y-BOCS score), whereas sexual abuse correlated with severity of obsessions but not compulsions. The authors point to the need for future longitudinal cohort studies to assess confounders such as the genetic variation and gene-environment interaction and to clarify a putative causal relationship between CM and OCD symptomatology.

The developmental subtype of OCD (early or pediatric onset OCD), in contrast to a later onset (adult) subtype, was addressed in a review by Geller et al.. The authors presented, in detail, the differences between pediatric and adult-onset OCD regarding familial patterns, comorbid disorders, phenotypic presentations, etiologies, neurocognitive and neuroimaging findings, treatment and outcome. They concluded that despite the body of evidence supporting the notion of a “developmental” pediatric subtype of OCD, additional translational and genetic studies are needed to clarify how the rapid development throughout the pediatric years, and the corresponding neuronal maturation, affect the presentation and research findings in youth and adults affected by the disorder.

## Genetics

Balachander et al. report on the familial aggregation of symptom dimensions among 330 first-degree relatives affected with OCD in 153 multiplex families. They observed a high concordance of OCD symptom dimensions within families, with sex-concordant dyads showing higher correlations than discordant ones for all the symptom dimensions, particularly checking, washing and arranging. They argue for the inclusion of symptom dimensions as key parameters in genetic and neurobiological studies in OCD in order to facilitate discovery of reproducible genetic and neuroimaging signatures of the disorder.

A review by Szejko et al. presents a comprehensive overview of the available Big Data resources for the study of OCD pathogenesis in the context of genomics. It is well accepted that, from the point of view of genetics, OCD is a highly heterogenous disorder, which is also reflected in the diverse clinical phenotypes and variable responses to treatment. Therefore, tools aimed toward developing personalized diagnostic and therapeutic approaches in OCD are much needed. Challenges like including more diverse populations and adopting harmonized methodology across studies need to be considered as the field moves forward. For now, studies targeting the genomics of OCD remain of great importance to unravel the complex genetic architecture of OCD. They are expected to contribute to find pathophysiological pathways involved in the occurrence of OCD and to guide treatment, especially in the context of personalized medicine.

## Neuroimaging

Murayama et al. investigated cerebellar-cerebral resting-state functional connectivity (rsFC) in unmedicated patients with OCD and healthy controls (HC) by determining seed ROIs in the cerebellum (related to the default mode network (DMN), the central executive network (CEN), the affective-limbic and motor networks) and verifying their rsFC to whole brain voxels. They report a significantly increased functional connectivity between the right lobule VI and the left precuneus in the OCD group as compared to HC. Based on the evidence that the precuneus is associated with higher-order cognitive processes and is one of the brain regions involving the DMN, the authors suggest that aberrant rsFC might occur not only in cerebral regions, but also in cerebellar-cerebral regions in OCD. In addition, they speculate that the increased rsFC between lobule VI, which has resting functional connectivity to the CEN, and the precuneus might relate to interference with the function of the DMN and involve the cognitive dysfunction in OCD. Of note, there was no correlation between the altered rsFC and obsessive-compulsive symptom severity. In face of this finding, the authors propose that the aberrant rsFC between the cerebellum and the DMN is not directly associated with the obsessive-compulsive symptoms (disease state marker), but a trait marker of OCD.

Computational models of OCD were addressed in a mini-review by Szalisznyó and Silverstein. In this approach, behavior and its neural correlates are quantitatively analyzed and computational models are developed to improve understanding of disorders by comparing model predictions to observations. The review covered mechanistic dynamical systems approaches, machine learning techniques (supervised and unsupervised models, reinforced learning), and Bayesian model selection frameworks. The authors related the modeling evidence and results to diagnostic procedures, co-morbid states, and therapeutical consequences in samples of patients with OCD. For clinicians with a traditional medical background, used to a phenomenologically based thought process, it may be challenging to follow this novel perspective. On the other hand, such dynamical approaches tend to develop in the near future, contributing to a more precise understanding of psychiatric illness and, hopefully, more personalized treatments.

## Neuropsychology

An original study by Ren et al. conducted in 46 Chinese adult patients with OCD and 45 healthy controls matched for age, sex and education aimed to add diversity to the neuropsychological studies in OCD, mostly represented by Western samples. The authors chose five tests of the Cambridge Neuropsychological Test Automated Battery (CANTAB) to evaluate response inhibition, spatial working memory, planning, and cognitive flexibility, along with testing basic learning and visual recognition memory. Unexpectedly, no significant patient-control differences were observed in the performance of any tests, as well as no significant differences in cognitive performance involving basic learning and memory. Moreover, within the patient group, no significant performance differences were detected between patients with mild or severe OCD symptoms. Methodological limitations that may have contributed to these results include the lack of control for variables that could be related to test performance, like socioeconomic status, medication status, or intelligence and the small sample size.

Ma et al., at Yale University, investigated the mechanisms associated with the difficulty in decision making often seen in individuals with OCD, by examining value-guided choice in OCD. The authors utilized a novel task, in which two types of decision are tested in parallel, using the same individually calibrated sets of visual stimuli (Perceptual and Value-based decision-making task, PVDM). Participants with OCD were compared to age and IQ-matched controls. Interestingly, a gender-dimorphic effect was observed, where decision formation was altered in OCD, but only in males, who were more cautious and less effective in evidence accumulation than male controls, and less likely than controls to adjust the process of evidence accumulation across decision contexts.

An investigation of the effects of stress and obsessive-compulsive symptoms on emotion regulation was investigated by Ferreira et al. in participants with OCD and healthy controls utilizing self-reported measures of stress levels, obsessive-compulsive symptoms, and emotion reappraisal and suppression skills. Their results revealed that elevated stress values predicted increased scores for suppression and decreased scores for reappraisal, with the reliance on suppression strategies and the difficulty in using reappraisal approaches better explained by stress levels and not directly explained by obsessive-compulsive symptoms. Although these findings need replication in untreated patients, they suggest a therapeutic potential for incorporating stress as a target in the psychotherapy for OCD.

To conclude this topic, Kashyap and Abramovitch provide us with an updated overview of the neuropsychological literature in OCD and present recommendations for future research in the field. The available evidence shows common findings across studies, indicating deficient test performance across cognitive domains with small to medium effect sizes. However, results remain inconsistent and heterogeneous, while multiple attempts to identify moderators that may account for such variability (like symptom severity, age at onset, medication status and comorbid conditions), have been unrewarding. The authors highlight less studied potential moderators that could exert an impact on neuropsychological findings in OCD, like the assessment of motivational and metacognitive factors related to performance, which is not part of standard neuropsychological research. The authors recommend that future research consider state/trait personal variables that may impact test performance in OCD, which may also increase interpretive power, and goodness-of-fit with psychopathological models. In addition, in order to increase the ecological validity of neuropsychological testing, the authors recommend that researchers address the definition of cognitive impairments and carefully select tests and outcome measures, incorporating the assessment of everyday function and utilizing the verisimilitude approach, incorporating tests that mimic the demands of real-life situations, instead of focusing solely on tests that may be correlated with real-life functions. Self-report systems tapping into real-life functions related to cognitive domains would also be of added value. They conclude by pointing that the coming efforts may need to be broader, by investigating the role of other factors impacting cognitive dysfunction; deeper (e.g., explore tests and constructs in relation to neuropsychological methods, clinical, and functional correlates), and finer (e.g., undertake more nuanced investigations of test performance), in order to advance the field.

## Phenotype

Apart from obsessions and compulsions, patients with OCD may present with symptoms pertaining to the sensorial domain. Bragdon et al. provide an interesting review on interoceptive processes in OCD (interoceptive accuracy, interoceptive sensibility and interoceptive awareness). Interoceptive sensibility appears to be the most consistently abnormal in OCD. For example, self-reported data suggest positive associations of the symmetry/ordering dimension with awareness of sensations and negative appraisal of internal sensations, pointing that interoceptive dysfunction may be relevant to this specific clinical presentation. In addition, neuroimaging investigations demonstrate the involvement of key interoceptive regions like the insula in the pathophysiology of sensory phenomena, urges-for-action, and disgust. Further knowledge on the relationship of interoception and related core OCD features could contribute to improving therapeutic outcomes.

Reports on the sexuality of patients with OCD are limited. A study by Dèttore et al. investigated the role of attachment styles and contamination symptoms as moderators of the relationship between gender and sexual arousal processes amongst patients with OCD according to the Dual Control Model. Their preliminary findings show that the relationship between gender and sexual arousal processes might be moderated by attachment styles and contamination symptoms. The authors suggest that sexual arousal should be more carefully evaluated during the clinical assessment of patients with OCD, and that gender-based effects of attachment styles and contamination symptoms should be taken into account to allow for personalized treatment planning.

The impact of the COVID-19 pandemic on OCD is addressed by Zheng et al., who report on the prevalence of OCD among residents in Wuhan 3 months after lifting the quarantine in the first outburst of the pandemic. The prevalence of OCD was 17.93%, with the most common obsessions being miscellaneous, aggressive and contamination, whereas miscellaneous, checking and cleaning/washing/repeating were the most common compulsions. Being single and a student, having a positive family history of OCD and other mental disorders, the presence of psychiatric comorbidity, and longer sleep latency were predictors of OCD in this specific situation.

## Comorbidity

A systematic review and meta-analysis on the lifetime comorbidities in OCD across the lifespan, by Sharma et al., covered the literature from the past 30 years. The authors adhered to the Preferred Reporting Items for Systematic Reviews and Meta-Analyses (PRISMA) guidelines, studies were clinic-based and reported original findings on individuals with OCD, evaluated with standardized diagnostic interviews. A pooled sample of more than 15,000 individuals yielded a comorbidity rate of 69%. Among children, anxiety disorders prevailed, whereas mood disorders prevailed in adults. Neurodevelopment disorders, specifically tic disorders and ADHD, were similarly prevalent among pediatric and adult samples. Personality disorders were prevalent in about 35% of the pooled sample from studies on adults with OCD, being OCPD the most common (17%), followed by anxious-avoidant (9%) and borderline personality disorder (9%), highlighting the need to include personality disorders assessment in the clinical evaluation of adults presenting with OCD. Among demographic factors, males presented higher rates of comorbidity with any psychiatric illness than females. Both the age of OCD onset and the age at assessment influenced the comorbidity profile and, in this way, are expected to also impact treatment. The collection of findings represents a relevant contribution to the need for more comprehensive clinical evaluation and management planning for those suffering with OCD.

An original study by Nicolini et al. aimed to compare the prevalence of use and dependence on cannabis in individuals with obsessive-compulsive symptomatology (OCS) with that of individuals with psychosis, depression, and anxiety symptoms, and to explore the association between genetic risk and use in 13,130 individuals evaluated in the second stage of the 2016 National Survey of Drug, Alcohol, and Tobacco Use (Encodat 2016). Genetic analysis (polygenic risk scoring-PRS) was available for a subsample of 3,521 individuals. Obsessive symptomatology had a 7.2% and compulsive symptomatology 8.6% prevalence. The proportion of individuals with OCS who had ever used cannabis was 23.4%, and of those with cannabis dependency was 4.3%, a figure higher than that in individuals with hypomania (2.6%), anxiety (2.8%) and depression (2.3%) and lower than in individuals with psychosis (5.9%). Individuals with OCS who reported using cannabis had an increased genetic risk for cannabis dependence but not for OCD. The authors hypothesize that the use of the PRS for cannabis dependence could be useful in predicting which individuals are at high risk, and to determine whether pharmacological treatment based on THC derivatives would be useful, or would exacerbate obsessive-compulsive symptomatology.

## Treatment

Zaboski et al. provide us with a narrative review on electroencephalography (EEG) correlates and predictors of treatment response in OCD. Most studies included medication or combined medication and CBT treatment, and one study looked at a small sample of subjects submitted to DBS. Initial studies addressing Error-Related Potentials using response inhibition tests found, for the Flanker test, that Error-Related Negativity (ERN) behaved more like an OCD-associated trait than a state-dependent correlate of symptomatology. In contrast, the Stroop test changed with treatment in another study. The P300, another ERP component, correlates with attention allocation and working memory while one is processing salient information. The P300 is elicited using an auditory oddball paradigm, where repetitive sounds are infrequently interrupted by a variant sound to which the participant must respond. Studies employing this paradigm before and after OCD treatment have reported conflicting results, with a tendency to consider that P300 amplitude and frequency differ at baseline in patients relative to controls, but only the amplitude changed at post treatment. Small studies on EEG complexity measures and individual oscillatory markers, along with limitations in identifying where in the brain the measured oscillatory signals arise, shall benefit from the recent advances in EEG data processing, such as techniques for band-specific source localization, allowing for more complex and efficient analyses and better source localization. Such approaches should help us to advance the knowledge on brain correlates of treatment response in OCD at the level of regions, networks, and frequency patterns.

The integrity of the white matter (WM) at pre and post treatment with concentrated exposure and response prevention (ERP) was investigated by Brecke et al.. They examined 32 patients with OCD and 30 matched healthy controls and searched for changes in the WM after a four-day treatment with concentrated ERP. The regions of interest (ROI) were the sagittal striatum, the posterior thalamic radiation and the cingulum, and longitudinal analyses were performed after 3 months of treatment completion. Despite a high rate of remission (77%), the authors did not find significant differences between groups in the various WM parameters before treatment, nor significant group by time effects in any of the ROI. Baseline FA measures were not associated with treatment outcome.

The maintenance of gains after inpatient intensive treatment for adults with treatment refractory OCD after 1 year post discharge was investigated in the UK by Nadeem et al., with encouraging findings. A hundred and thirty patients were admitted with severe OCD, with an average Y-BOCS total score of 36.9 at admission. After intensive treatment comprising pharmacologic optimization, individualized exposure programs and group therapy sessions focusing on facing up to fear and activities of daily living, the mean YBOCS total score dropped to 23.4 (36% mean reduction). Eighty percent of the sample remained available until the 12th month follow-up, when the maintenance of gains revealed stable around a 40% improvement.

The role of adherence in the success of intensive behavioral treatment was studied by Tjelle et al., in a sample of 42 patients that received the Bergen 4-day format concentrated exposure/response prevention treatment. Adherence was measured with the Exposure and Response Prevention Adherence Scale (PEAS), rated both by patients and therapists, after the second and third day. Treatment outcome, assessed at 3-month follow-up, consisted of a 71.4% remission rate, associated with high adherence scores, which the authors suggest could be related to the concentrated format making it easier for the patient to adhere to treatment given the short time period, and that patients selecting intensive treatment are more able to sustain motivation during this brief period. Importantly, besides improvement of OCS, adherence rates correlated also with improvement of anxiety and depression, well-being, and work- and social functioning.

Clinical, sociodemographic and psychosocial predictors of the need for intensive treatment were investigated by du Mortier et al. in 419 patients with OCD using 6-year longitudinal data of the Netherlands Obsessive Compulsive Disorder Association. Being single, more severe comorbid depression, use of psychotropic medication, and a low quality of life predicted intensive treatment in the following 2 years. The authors recommend that therapists stay aware of such predictors in order to optimize first-step treatments and prevent the necessity of intensive treatment. Besides, the predictors might be used to tailor intensive treatment to the characteristics of patients involved. Examples include working on comorbid depression and personal goals in treatment, in addition to working on OCD, and providing extra support in treatment for patients who need it and to adjust treatment to impairments due to morbidity and/or a low quality of life.

The feasibility and efficacy of a flexible dose regimen of intensive CBT for youth with OCD was addressed by Selles et al. in a randomized pilot study. The authors also compared outcomes across home and hospital setting delivery. The results confirmed the efficacy of the intensive CBT program, with significant improvement in OCD and more modest benefits in comorbid symptoms and quality of life. Observed differences in treatment session utilization levels across participants suggest that flexibility in treatment dosing is desirable and useful in optimizing levels of care based on individual need. Treatment was rated highly by participants. The authors concluded that adjusting the amount of treatment provided based on patient need/preference is feasible and allows for flexible allocation of resources. In addition, although treatment setting was not found to have a major impact on outcomes, treating patients within their home environment may offer some additional benefits in generalizability and maintenance of gains as well as youth satisfaction.

Another attempt toward a more individualized approach to treatment is described in the original article by van Steenwijk et al.. They examined the relation between the performance on a pre-treatment behavior approach test on willingness and ability to fully engage in exposure/response prevention (BAT) and symptom change after 12 weeks of intensive residential treatment (IRT). Although the performance on the BAT was significantly associated with symptom change after IRT, its effect-size was insufficient to justify transforming the BAT in its current fashion into a clinically useful instrument for indicating which treatment and treatment-setting is most promising for the individual patient. The authors discuss the need for tools that can differentiate between the patients who do profit from IRT and the ones who do not or are in need of more extensive preparation trajectories.

Taken together, the manuscripts within this Research Topic cover a broad range of themes related to the current diagnostic challenges and the search for personalized treatment in OCD across the lifespan. There is still a long way to go, as pointed by the authors in the numerous directions for future research in each theme. Nevertheless, this initiative demonstrates how the efforts across the globe adopting rigorous methodological standards can achieve continuous progress toward a deeper understanding of OCD in its many facets, and positively impact clinical practice in addressing the unmet needs of patients throughout their lifetime.

## Author contributions

RGS has prepared the first draft of this editorial. OAH, CL, YCJR, ECM, and HBS have revised the first draft and contributed to the final version of the manuscript. All authors contributed to the article and approved the submitted version.

## Conflict of interest

RGS has received consultancy honoraria from Lundbeck and research grants from Conselho Nacional de Desenvolvimento Científico e Tecnológico (CNPq). OAH has received consultancy honoraria from Lundbeck. HBS has received research support from an industry-sponsored clinical trial from Biohaven Pharmaceuticals, royalties from UpToDate Inc., from Cambridge University Press. The remaining authors declare that the research was conducted in the absence of any commercial or financial relationships that could be construed as a potential conflict of interest.

## Publisher's note

All claims expressed in this article are solely those of the authors and do not necessarily represent those of their affiliated organizations, or those of the publisher, the editors and the reviewers. Any product that may be evaluated in this article, or claim that may be made by its manufacturer, is not guaranteed or endorsed by the publisher.
